# Transcriptional Activation of *c3* and *hsp70* as Part of the Immune Response of *Acropora millepora* to Bacterial Challenges

**DOI:** 10.1371/journal.pone.0067246

**Published:** 2013-07-04

**Authors:** Tanya Brown, David Bourne, Mauricio Rodriguez-Lanetty

**Affiliations:** 1 Department of Biological Sciences, Florida International University, Miami, Florida, United States of America; 2 Australia Institute of Marine Sciences, Townsville, Queensland, Australia; University of New South Wales, Australia

## Abstract

The impact of disease outbreaks on coral physiology represents an increasing concern for the fitness and resilience of reef ecosystems. Predicting the tolerance of corals to disease relies on an understanding of the coral immune response to pathogenic interactions. This study explored the transcriptional response of two putative immune genes (*c3* and *c-type lectin*) and one stress response gene (*hsp70*) in the reef building coral, *Acropora millepora* challenged for 48 hours with bacterial strains, *Vibrio coralliilyticus* and *Alteromonas* sp. at concentrations of 10^6^ cells ml^-1^. Coral fragments challenged with *V. coralliilyticus* appeared healthy while fragments challenged with *Alteromonas* sp. showed signs of tissue lesions after 48 hr. Coral-associated bacterial community profiles assessed using denaturing gradient gel electrophoresis changed after challenge by both bacterial strains with the *Alteromonas* sp. treatment demonstrating the greatest community shift. Transcriptional profiles of *c3* and *hsp70* increased at 24 hours and correlated with disease signs in the *Alteromonas* sp. treatment. The expression of *hsp70* also showed a significant increase in *V. coralliilyticus* inoculated corals at 24 h suggesting that even in the absence of disease signs, the microbial inoculum activated a stress response in the coral. *C-type lectin* did not show a response to any of the bacterial treatments. Increase in gene expression of *c3* and *hsp70* in corals showing signs of disease indicates their potential involvement in immune and stress response to microbial challenges.

## Introduction

Coral reefs worldwide are in decline due to natural and anthropogenic stressors both at the local and global scales [1]–[4]. An emerging factor contributing to their decline is the outbreak of destructive diseases caused by bacteria, viruses, protozoa, or fungi that are observable in the field as lesions or bands of tissue loss [2], [5]–[7]. However, the causes of many coral diseases still remains unknown [2], [8], [9] due to a number of factors including inconsistent disease descriptions [10], complex host/agent interactions, challenging experimental systems, and the potential that many pathogens may not be cultured in the laboratory [2], [11]. Microorganisms may be normal components of the reef ecosystem, though altered environmental conditions may shift benign organisms to pathogenic roles via the expression of virulence factors [8]. The best studied example is the *Vibrio shiloi* infection of the Mediterranean coral *Oculina patagonica* where bacterial virulence is enhanced by increased seawater temperatures, resulting in coral bleaching [9], [12], [13]. Increases in seawater temperatures might also compromise the immune system in corals and therefore affect their ability to fight infections, particularly those corals that have undergone bleaching due to thermal stress [14]–[16]. It remains unknown whether environmental stress (e.g. increased water temperatures or eutrophication) taxes the coral host metabolically, thus increasing its disease susceptibility. A better understanding of the actual mechanisms employed by corals to fight and resist disease-causing agents and how host defense mechanisms are compromised by environmental factors is required to tease apart these complex interactions.

Though the current understanding of cnidarian and coral immunity is rudimentary [17], a number of studies have provided insight into how cnidarians protect themselves from infection [18], [19]. The combination of cellular and humoral factors utilized to respond to microbial challenges has been shown to vary from one organism to another. For corals, it has been documented that mucociliary activity [20], [21], skeletal biomineralization [22], antimicrobial activity [23]–[25], and melanization and phenoloxidase activity [16], [26] appear to play important roles in the defense against microbial infectious agents.

Furthermore some cnidarian genes homologous to innate immune genes from higher metazoans have been identified [27]–[33]. However, the functional involvement of these putative immune genes in cnidarian immunity has not yet been experimentally verified [17]. To avoid falling into the “homology trap” where gene homology is based on the wrong notion of concordance [17], [34], it is now imperative to start characterizing and confirming the functional role of putative immune homolog genes identified in the context of coral immune response. Of particular interest in this study are two putative coral immune genes: *c3* and *mannose-binding c-type lectin*, which have been shown to be involved in the immune repertoire of other biological model systems. Additionally a heat stress response gene, *hsp70*, was assessed due to the general importance of stress core genes in the response of organisms to a large number of biological and abiotic stressors [35].

The complement system is an important element of the complement cascade that has been well documented in higher metazoans and is involved in opsonization of pathogens, chemotaxis and activation of leukocytes, and direct killing of pathogens [36]–[38]. In some invertebrates such as in the horseshoe crab, *Carcinoscorpius rotundicausaa*, an opsonization role has been described for a homolog of complement C3-like protein [39]. In cnidarians, several *c3* homolog genes have been identified from transcriptome sequencing projects including the octocoral *Swiftia* [29], the scleractinian *Acropora millepora* [27], the sea anemones *Nematostella vectensis* [40], and *Aiptasia pallida* [32]. Despite this, the function and involvement of C3-like proteins in cnidarian immunity, remains untested.

Another important component of the immune repertoire in both vertebrate and invertebrate organisms are carbohydrate–based recognition receptors such as C-type lectins [36], [41]. C-type lectins bind to glycans and thus play a role in biological processes such as cell-cell adhesion, pathogen recognition, bacterial cell wall recognition, and phagocytosis [36], [41]. Identified lectin homologs in cnidarians contain extensive sequence variation, which may indicate the potential to bind bacterial pathogens [33]. For example, in the anemone *Nematostella vectensis*, 92 putative *c-type lectin* genes have been described [33]. However, the connection of many of these c-type lectin proteins with respect to the immune functional response of corals to microbial agents has not been established.

Heat shock protein expression increases after exposure to abiotic stressors including heat or cold challenges and biotic challenges during infection and disease development [42], [43]. The involvement of these proteins in the immune system has also been widely reported in higher metazoans showing a connection to the activation of the innate complement system [44]. For instance, human HSP70 activates the Toll/IL-1 receptor signaling pathway during a highly inflammatory response [42], [43]. Moreover, accumulation of Hsp70 following sub-lethal thermal treatments in the invertebrate *Artemia franciscana* appears to provide protection against subsequent pathogen challenges [43], [45]. The involvement of coral *hsp70* has been clearly shown in the response to thermal challenges [31], [46], [47]; however its implication in coral immunity is still unknown.

This study investigated the transcriptional changes of the three genes of interest *c3*, *mannose-binding c-type lectin*, and *hsp70* from the widely distributed Indo-Pacific coral *Acropora millepora*. Challenges were conducted using the identified coral bacterial pathogen, *Vibrio coralliilyticus* and a potential pathogen, *Alteromonas* sp. The actual functional involvement of these putative immune genes in corals during the defense response to infectious agents has not been experimentally verified and it is imperative to characterize and confirm these functional roles in the context of coral immune response [17].

## Materials and Methods

### Coral Collection and Acclimation

Two adult *A. millepora* coral colonies were collected from reefs around Orpheus Island (18°37′06″S 146°29′37″E) in the inner central section of the Great Barrier Reef. Coral nubbins 4–5 cm in length were fragmented from the adult coral colonies and acclimatized for five weeks in outdoor 5000-L aquaria under natural light conditions at the Australian Institute of Marine Sciences (Townsville, Australia). Coral nubbins were then placed in experimental aquarium tanks and allowed to recover from the mechanical manipulation for eight days prior to experimental treatments. Corals in the experiment were collected under the permit number G09-30237 emitted by the Great Barrier Reef Marine National Park Authority, Australia.

### Bacterial Strains and Culture Preparation

Two bacterial strains were used in this study: *Vibrio coralliilyticus* strain LMG 23696, previously identified as a coral pathogen isolated from Nelly Bay Magnetic Island, Australia [48], and an *Alteromonas* species isolated from *A. millepora* corals also sampled from Magnetic Island. *Vibrio coralliilyticus* has been implicated as one of the causes of white syndrome disease in Acropora corals [11], [48] while *Alteromonas* spp. have been correlated with disease and also a normal resident of the mucus layer [49], [50], [51], [52], [53] and skeleton [53], [54]. The 16S rRNA gene sequences for each strain are deposited in the GenBank database under the following accession numbers, EU372917 and GU903232 respectively.

Bacterial strains were recovered from glycerol stocks and inoculated into the general heterotrophic bacterial medium, Marine Broth-2216 (Difco, USA) and grown to end logarithmic phase at 27°C with shaking (150 rpm). Cultures were centrifuged at 5,000g for 10 minutes, washed and resuspended in phosphate buffered saline (PBS). This process was repeated three times to remove residual culture media. The cells were prepared to a final concentration of 1×10^9^ cells ml^-1^ in PBS. Bacterial cell density was determined by counting colony-forming units (CFU; described by Sussman et al [48]) and by constructing a cell density calibration curve of absorbance (595 nm) vs. CFU.

### Experimental Design of Bacterial Challenge Experiments

Three acclimated coral nubbins were placed in each of nine replicated 5-L aquarium tanks. Coral nubbins from colony 1 were placed in three tanks and challenged with *Vibrio coralliilyticus*. Nubbins from colony 2 were placed in three other tanks and challenged with *Alteromonas* sp. Finally three tanks were allocated for control corals, each containing 6 nubbins (three from each colony). Two separate experiments were conducted under this experimental design, with the treatment group compared to the control nubbins originating from the same colony. Each 5-L aquarium was inoculated with the relevant bacterial strain to a final cell concentration of 1×10^6^ cell ml^−1^ in each tank. Bacteria were not added to control tanks, though a similar volume of PBS was added since it was used to wash the bacteria before inoculation in the treatments. The aquaria were operated as a closed system: seawater was not replaced for the duration of the 48-hour experiment to avoid potential cross-contamination and release of bacteria. However, aeration was maintained in the tanks to provide water movement. Temperature loggers were deployed in the tanks to assess the temperature fluctuation during the entire course of the 48-hour experiment. Visual observations of the coral nubbins were conducted every hour for the first 12 hours and then every six hours until the end of the experiment.

Nubbins were collected 6 and 24 hours after the bacterial inoculation. Nubbins could not be collected at 48 hours after bacterial inoculation since the *Alteromonas* sp. inoculated nubbins had very little viable tissue associated with them. At each sampling time, one coral nubbin from each replicate tank was collected and immediately snap frozen in liquid nitrogen, and stored at −80°C until sample processing.

A second bacterial challenge experiment was repeated which corroborated the visual results of the effect of bacterial inoculation on coral nubbins. All nubbins from this repeat experiment were fragmented from a single colony and not acclimatized to the light conditions in the outdoor aquarium facility prior to the experiment. All other aspects of the experiment were identical to the first though samples were not collected for subsequent molecular analysis.

### PSII Quantum Yields (F_v_/F_m_) of Coral Samples

The photosynthetic efficiency (PSII quatum yield) of the associated symbiotic dinoflagellates (*Symbiodinium*) from all coral nubbins (treatments and control) were assessed using a Maxi imaging-pulse-amplitude-modulation (iPAM) fluorometer (Walz, Germany) 48 hours after bacterial inoculation. Coral nubbins were placed in darkness for 20 min and then exposed to a saturation light pulse (Gain = 1–2, Intensity = 1–2, Saturation Pulse = 7) using the iPAM. The dark adapted PS II quantum yields were calculated by using the formula: F_v_/F_m_ = (F_m_-F_0_)/F_m_, where F_m_ = maximal fluorescent yield, and F_0_ = Dark fluorescent yield.

### RNA Isolation, cDNA Preparation, and Gene Expression Assays by Quantitative PCR

Frozen coral nubbins were pulverized into powder using a French press under ultra-freezing conditions (∼ −120°C). Total RNA was extracted from approximately 200 µg of frozen pulverized coral tissue samples using the RNeasy Plant Mini Kit (Qiagen, Valencia, CA) according to the manufacturer’s protocol. Concentrations of total RNA were determined using the NanoDrop ND 1000 UV-Vis Spectrophotometer (NanoDrop Technologies Inc, Wilmington DE). Integrity of the samples was checked on MOPS denaturing RNA gels (Embi Tec, San Diego, CA). Total RNA (250 ng) was reverse transcribed to cDNA using the QuantiTect reverse transcription kit (Qiagen, Valencia, CA) according to the manufacturer’s protocol.

Quantitative real time PCR (qPCR) was performed on the three genes of interest, *c3*, *c-type Lectin*, and *hsp70* using a Rotor Gene Q1 cycler (Qiagen, Valencia, CA). Several other putative coral immune genes such as *TLR4-like*, *TLR-23-like*, and several isoforms of C-type lectins were also initially explored but no adequate PCR oligonucleotide were developed. One microliter from each of the reverse transcription reactions was used along with the Rotor Gene SYBR Green PCR master mix (Qiagen, Valencia, CA) to carry out the qPCR according to the manufacturer’s protocol. The primers used to amplify each gene are shown in Table 1. Primer concentrations were optimized for each of the primer pairs, resulting in the use of 1 µM for *c3* and *hsp70*, and 0.5 µM for *c-type lectin*. Additionally, two internal control genes (ICGs), *actin* [55]–[57] and *ribosomal protein 12* (*rpl12*) [56], [58], were run simultaneously for normalization of data using a concentration of 1 µM for both the forward and reverse primers. Reactions for each gene of interest and ICGs were performed in triplicate. The comparative delta Ct method was used to correct for PCR efficiency and determine relative quantities of the transcript.

**Table 1:** Forward and reverse primers used to amplify the following genes of interest (GOI), including internal control genes (ICG), in Q-RT-PCR assays.

Gene	PCR Product		Primer Sequence (5′ to 3′)
*c3*		96 bp	For: GTGAAGGTGGAACCAGAGGA
			Rev: GAACCGGAAGTGATTGTCGT
*c-type lectin*		230 bp	For: CAGGTCTGGATCGGACTCAT
			Rev: CATGTCCAGTGGTTGTACGC
*hsp70*		128 bp	For: GAGCCCTCAGTAACCAGCAC
			Rev: CATTGTGGAGCGGAAAAGTT
*rpl12* (ICG)		150 bp	For: CAAGGCAACACAAGACTGGA
			Rev: CTTGCGATCTTGGTGGTT
*Actin* (ICG)		113 bp	For: CTCTTCCCCATGCCATCTTA
			Rev: TTGATGTCTCGCACGATCTC

### RNA Profiling of Coral Associated Bacterial Communities using Denaturing Gradient Gel Electrophoresis

Total RNA was extracted from coral nubbins collected at 24-hours post bacterial challenge using methods described previously. RNA samples (100 ng) were reverse transcribed using the QuantiTect reverse transcription kit (Qiagen) with 1 µM of the modified bacterial specific primer 907R (CCTACGGGDGGCWGCAG) [59]. After reverse transcription, samples were amplified in 50 µl reactions using GoTaq Green Master Mix (Promega, Madison, Wisconsin, USA) with 2.5 mM MgCl_2_, 0.25 µM 907R, and 0.75 µM 341F-Clamp (CGCCCGCCGCGCCCCGCGCCCGTCCCGCCGCCCCCGCCCGCCTACGGGDGGCWGCAG) [59]. The PCR program was as follows: 5 minute initial denaturation at 95°C, followed by 35 cycles of 95°C for 30 seconds, 51°C for 60 seconds, and 72°C for 60 seconds, and a final extension for 7 minutes. The PCR products were run on DGGE using a 6% acrylimide denaturing gradient gel (30–65% gradient) for 14 hours at 97 volts at a constant temperature of 60°C.

Bands of interest were excised from the gel and incubated at room temperature for 24 hours in 30 µl nuclease–free water followed by recovery of the DNA by ethanol precipitation. Samples were resuspended in 30 µl nuclease-free water and 1 µl of the sample was used in a 25 µL PCR reaction using GoTaq Green Master Mix (Promega, Madison, Wisconsin, USA), 0.25 µM of both 341F and 907R that did not contain a GC clamp. The PCR cycle was as follows: 5 minute initial denaturation at 95°C, followed by 35 cycles of 95°C for 30 seconds, 51°C for 60 seconds, and 72°C for 60 seconds, and a 7 minute final extension. PCR products were directly sequenced by the DNA Analysis Facility at Yale University (New Haven, Connecticut, USA) using the 907R primer. Recovered sequence identity was assessed using a BLAST comparison in GenBank and a check for chimeras was carried out using Greengenes Bellepheron database [60].

### Statistical Analysis

Data that were not homoscedastic were transformed prior to downstream analysis. Statistical analyses of the gene expression data were performed on each of the genes of interest using the relative copy numbers normalized to the geometric mean of the two internal control genes following the approach by Vandesompele et al [61] and Rodriguez-Lanetty et al. [56]. Significant differences in gene expression among treatments (bacterial treatments vs. control) and across time (6 and 24 h) were tested with a two-way ANOVA (SAS, Cary, NC). Comparisons were only executed within bacterial treatments (i.e. within the *Alteromonas* treatment and separately within the *V. coralliilyticus* treatment) since separate colonies were used in each treatment.

DGGE gel images were digitized using Gel2K [62] in order to create a presence/absence matrix. This data was analyzed with a correspondence analysis and was performed using the Vegan package for the R environment [63], [64].

## Results

### Response of Acropora Millepora Corals to Bacterial Challenge

**Figure 1:**
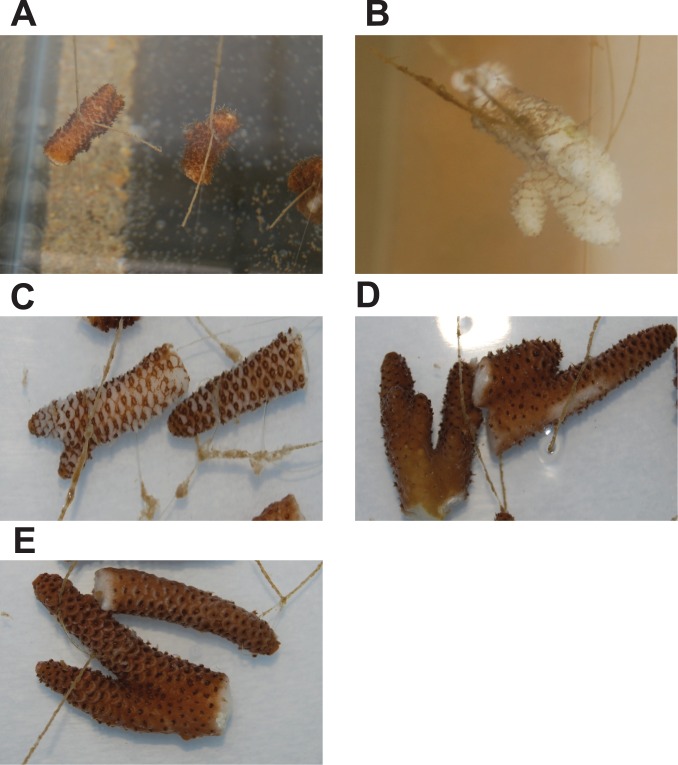
Photographs of *Acropora millepora* coral nubbins from the first experiment at 48 hours after bacterial inoculation. A: corals challenged with *Vibrio coralliilyticus*; B: corals challenged with *Alteromonas* sp. Photographs of coral nubbins from the second experiment at 48 hours after inoculation with *Alteromonas* sp. at different concentrations. C: 10^6^ CFU/ml; D: 10^5^ CFU/ml, and E:10^4^ CFU/ml.

No bleaching or visible lesions were observed on *A. millepora* nubbins challenged with *V. coralliilyticus* (1×10^6^ bacteria ml^−1^; Fig. 1A) or the control treatments throughout a 48-hour bacterial challenge experiment. In contrast, coral nubbins challenged with *Alteromonas* sp. (1×10^6^ bacteria ml^−1^), displayed signs of bleaching and lesions in the coenosarc tissue (tissue between polyps) after 24 hours. By 48 hours, most of the coenosarc and polyp tissue was degraded in all replicate coral nubbins in this treatment (Fig. 1B). A repeated experiment resulted in the same results with all coral nubbins challenged with the *Alteromonas* sp. showing tissue necrosis (Fig. 1C), while *V. coralliilyticus* challenged and control nubbins displayed no signs of lesions (Figure S1). However, coral nubbins challenged with lower concentrations of *Alteromonas* sp. (1×10^5^ and 1×10^4^ bacteria ml^−1^) did not show signs of lesions and/or bleaching (Fig. 1D-E).

**Figure 2:**
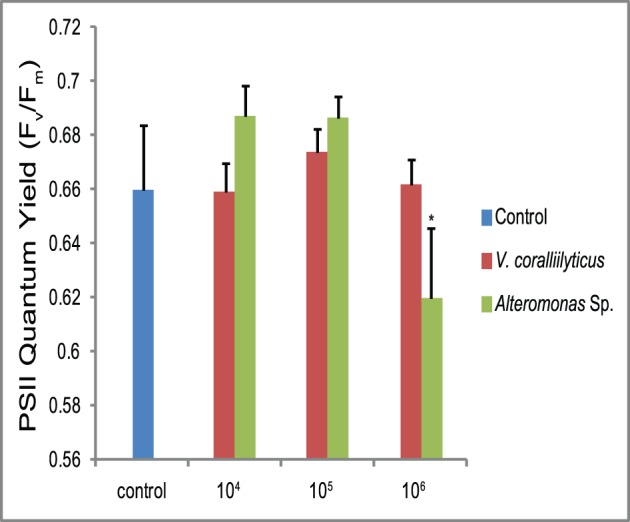
Dark-adapted PSII quantum yield (F_v_/F_m_) of *Symbiodinium* associated with coral nubbins after 48 hours from the bacterial inoculation during the second set of experiments. Error bars indicate standard deviation of the mean. (*) indicates significance (p<0.05) between that treatment and the control.

Photosynthetic efficiency (PSII quantum yield; *F_v_/F_m_*) of symbiotic dinoflagellates (*Symbiodinium*) was significantly reduced (one-way ANOVA, p<0.01; Fig. 2) following challenge by *Alteromonas* sp., confirming a bacterial effect on the photophysiology of the symbiotic dinoflagellates associated with the coral nubbins. In contrast, nubbins challenged with *V. coralliilyticus* did not show a significant difference in photosynthetic efficiency, indicating that *Symbiodinium* in these coral nubbins were not compromised.

**Figure 3:**
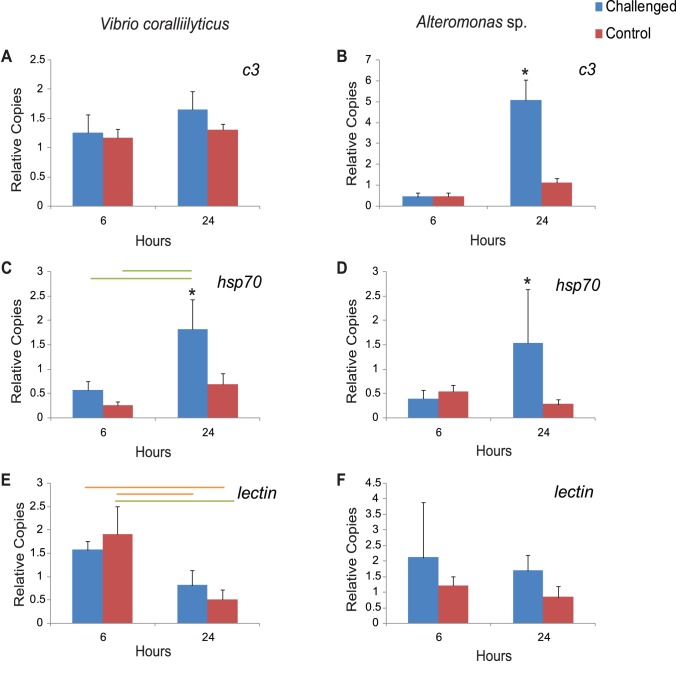
Relative transcriptional expression of the three genes of interest from *Acropora millepora* at 6 and 24 hours after inoculation with either *V. coralliilyticus* or *Alteromonas* sp. A–B: *c3*; C-D: *hsp70*; E–F: *c-type lectin*. The Q-RT-PCR data for these genes were normalized using internal control genes (ICGs) indicated in Table 1. Error bars indicate standard deviation of the mean. (*) indicates significance between treatment and control at the indicated hour. Green bar indicates significance at p<0.01 and orange bar indicates p<0.05.

No changes of *c3* gene expression was detected (two-way ANOVA, p = 0.146; Fig. 3A) for coral nubbins challenged with *V. coralliilyticus*. However, an increase of *hsp70* gene expression was detected at 24 h in the challenged corals compared to the controls (ANOVA p = 0.0149; Fig. 3C). The gene expression of the *c-type lectin* in the *V. coralliilyticus* experiment did not show a response to the bacterial challenge (2 way ANOVA p = 0.9449) but did decrease significantly between 6 and 24 hours for the controls (ANOVA, p = 0.0068; Fig. 3E). For coral nubbins challenged with *Alteromonas* sp., which showed signs of tissue lesion and disease, a significant increase of gene expression of *c3* was detected from 6 hours to 24 hours (two-way ANOVA, p = 0.002, Tukey, p<0.0001; Fig. 3B). In addition, the increased *c3* gene expression at 24 hours in this *Alteromonas* sp. bacterial challenge was significantly higher than controls (Tukey, p<0.0001; Fig. 3B) at the same time point. Similar to the *c3* gene expression, the transcriptional profile of *hsp70* in *Alteromonas* sp. challenged nubbins was significantly higher at 24 hours than controls (ANOVA, p = 0.0159; Fig. 3D). Additionally, no significant changes were observed in the gene expression profile of control coral nubbins at 6 and 24 hours for *hsp70* (ANOVA, p = 0.3744). The transcriptional response of the *c-type lectin* was not affected by the bacterial challenge (2 way ANOVA p = 0.1532) when compared to the controls but did show a nonsignificant decreasing trend in expression from 6 to 24 hours (Fig. 3F).

### Coral Associated Bacterial Shifts during Challenge Experiments

**Figure 4:**
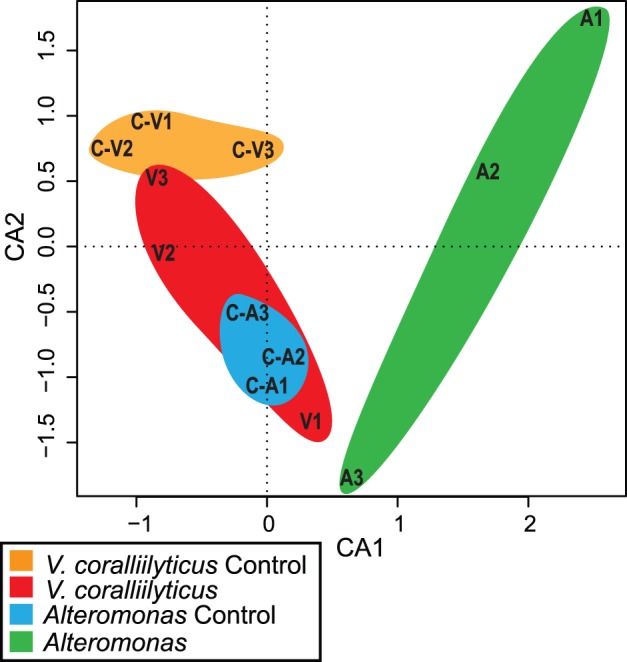
Correspondence analysis (CA) of bacterial 16S rDNA-DGGE banding patterns. CA1 accounts for 26.9% of the variation; CA2 accounts for 20.8% of the variation. C-V, control-colony 1 (orange); C-S, control-colony 2 (blue); V, *vibrio* treatment (red); A, *Alteromonas* sp. treatment (green).

Profiling of bacterial communities associated with coral nubbins using denaturing gradient gel electrophoresis (DGGE) combined with multivariate correspondence analysis of a presence/absence matrix for all observed bands, documented shifts in the bacterial community for *Alteromonas* sp. challenged corals (Fig. 4; Figure S2). The bacterial assemblages associated with the *Alteromonas* treatment clustered separately from all controls, differentiated mainly by the CA1 axis of the correspondence analysis, which explained 26.9% of the variation (Fig. 4). *V. coralliilyticus* treatments also clustered separately from its control by the CA2 axis which accounted for 20.8% of the variation. Interestingly, the bacterial community profile associated with the *V. coralliilyticus* treated nubbins was similar to the community profiles of control nubbins for the *Alteromonas* sp. treatment (Fig4). All bands that were able to be excised and sequenced from the DGGE are deposited in Genbank (accession numbers KC313998– KC314010). The retrieved sequences from both treatments were affiliated within the *Gammaproteobacteria* and profiles were consistent across all the samples, indicative of a stable microbial community. Sequences affiliated to the genus *Endozoicomonas* (BLAST identity: 96% sequence identity over 527 bp) were identified as one of the dominant members of both microbial communities. None of the dominant bands in the DGGE represented any species of *Vibrio* or *Alteromonas* sp, corroborating the lack of infection by *V. coralliilyticus* in the experiment. An unclassified sequence to the level of genus but also related to the order of Oceanospirillales (accession number: AB680857) was retrieved from only corals challenged with *V. coralliilyticus*.

## Discussion

This study compared the visual tissue disease development and the transcriptional response of several putative immune genes from *Acropora millepora* to challenge from two bacterial strains *V. coralliilyticus*, and an *Alteromonas* sp in two separate experiments. Transcriptional increases of *c3* and *hsp70* in the *Alteromonas* sp. challenged corals suggest the involvement of these genes in the immunological/defense response of the coral, *A. millepora* to microbial challenge. Corals challenged with *V. coralliilyticus* did not show visible signs of disease, however, an increase in *hsp70* suggests bacterial inoculation activates a coral host stress response and implicates heat shock proteins as a critical component in the early responses of corals to potential infectious agents. The response likely occurs prior to a strong host immune reaction and before visible signs of disease is apparent.

*C3*– like protein has previously been postulated to play a role in the innate immune system in cnidarians [27], [29]. However, this hypothesis has remained untested due to a lack of functional data. Findings from this study support the previous hypothesis since the expression of *c3* in *A. millepora* increased in response to *Alteromonas* sp. bacterial challenge. The increase in expression coincided with visual signs of disease in the coral nubbins at 24. To our knowledge, this is the first study to show transcriptional activation of a cnidarian C3–like protein gene in response to a live microbial challenge. A previous study in which lipopolysaccharide (LPS) was injected into *A. millepora* failed to show a significant response of *c3* to the treatment [65]. However, their experimental outcome could have been due to LPS only being one component involved in bacterial mediated virulence. This is corroborated in a study in *Drosophila* which showed that peptidoglycan – free LPS caused a seven-fold weaker response than a living Gram – negative bacterium [66]. This highlights the importance of using a live pathogen when screening for components involved in the immune response. The transcriptional upregulation of *c3* in response to microbial challenges reported here, coupled with previous molecular characterization showing similarity in functionally critical domain structures and amino acid residues of cnidarian C3-like protein homologs to those in higher metazoans [27], [29], [40] are consistent with a key role of C3-like protein in the immune response of corals. Additionally, it has previously been demonstrated that the transcriptional expression of *c3* is localized in the gastrodermal cells [27], the cells lining the gastrovascular cavity that interact with a vast number of microbes [67]. Further studies are now required to determine whether C3-like protein has a role in opsonization and pathogen recognition. Unlike the corals challenged with *Alteromonas sp*, *V. coralliilyticus* inoculated coral nubbins were visibly healthy after 48 hours, with no changes in *c3* expression and photosynthetic yield of *Symbiodinium* being observed. This suggests that corals were less compromised physiologically by the bacterial challenge with *V. coralliilyticus* and that the inoculation may not have activated a strong immune response.

Another novel finding from our study is the transcriptional upregulation of *hsp70* detected in both the *Alteromonas* sp. and *V. coralliilyticus* treatments. The expression of heat shock proteins in general, including Hsp70, have been shown to increase with heat stress in corals [31], [68]–[79]. Our findings suggest that changes in *hsp70* transcripts also occur in corals in response to microbial challenges, in the absence of thermal stress. Several studies conducted on the brine shrimp *Artemia* have already shown that heat-induced accumulation of Hsp70 appears to protect *Artemia* sp. from pathogenic infection by *Vibrio campbellii* [43], [80]. Recently, Baruah et al (2011) presented evidence that Hsp70 enhances resistance to pathogens by priming and enhancing the expression of the prophenoloxidase system. Prohaszka and Fust (2004) proposed that extracellular heat shock proteins may represent the ancestral danger signal of cell death or lysis-activating innate immunity [81]. In support of this hypothesis, human HSP70 has been implicated in the antibody-independent activation of the complement immune system, ultimately interacting with C3 [42]. Thus, in the *Alteromonas* sp. challenged nubbins, the increased expression of *hsp70* could be a downstream result of pathogen recognition and subsequent involvement in the immune activation of a C3– like protein in corals, though this hypothesis requires further experimental investigation. A different scenario was observed in *V.coralliilyticus* with a significant increase of *hsp70* transcripts, even when there was no visible sign of disease, but lack of expression of *c3*. In this case an increase of Hsp70 proteins following host/pathogen recognition was potentially sufficient to protect the coral, possibly by activating other constitutive components of the coral effector immune systems, such as the pro-phenoloxidase cascade. These findings highlight the potential importance of heat shock proteins, in particular Hsp70, as a core stressor protein useful in assessing coral health status.

Unlike the clear transcriptional response of *c3* and *hsp70* to bacterial challenges, the *c-type lectin* gene examined in this study did not show a differential change at the transcriptional level to bacterial treatments. C-type lectins are proteins well known to act as pattern recognition receptors enhancing pathogen removal through phagocytosis in invertebrates [41] and/or activation of the complement system cascade following pathogen recognition in higher metazoans [37]. Moreover, *c-type lectins* found in cnidarian genomes have been shown to have a highly variable substrate-binding region, suggesting that this domain may recognize a large range of pathogens [30]. The lack of a significant transcriptional response of *c-type lectin* to the bacterial challenges conducted in our study could be attributed to the timing of sampling. It is possible that we could have missed an early up-regulation of this gene within the first six hours after bacterial inoculation. Consistent with this rationale is the fact that Kvennefors et al (2010) documented a significant increase in expression of *millectin* after only 45 minutes post injection with LPS. After this initial peak, expression of the gene decreased gradually over the following twelve hours [65]. This could also explain the fact that we detected a decreasing trend, though not statistically significant, in the expression of *c-type lectin* from 6 to 24 hours in the bacterial treatments. Further studies are required to confirm whether or not the c-type lectin explored here plays a role in coral immunity.

*A. millepora* nubbins exposed to *V. coralliilyticus* did not exhibit detectable signs of lesions throughout the 48-hour experimental period. Pathogen infection trials are highly problematic due to complex host/pathogen interactions, which are very difficult to control in aquarium-based environments. One contributing factor is that *V. coralliilyticus* has not yet been conclusively identified as a pathogen of *A. millepora* and host specificity is important in coral infections. However, some experimental infections on coral juveniles suggest that *V. corallilyticus* can replicate the development of white syndrome disease in *A. millepora* [82]. *V. coralliilyticus* virulence has also been demonstrated to be water temperature dependent with virulence being activated at 27–29°C [12], [13]. A study investigating *V. coralliilyticus* infection of *Pocillopora damicornis* demonstrated no lesions for treatments at 25°C [83], temperatures similar to those used in the bacterial challenges of this study. Despite the fact that no disease developed during the *V. corallilyticus* treatment, this study focused on exploring the gene expression response of coral to the bacterial challenge itself. *Alteromonas* sp. challenged nubbins did show pronounced lesions within 24 hours of treatment. In corals, *Alteromonas* sp. has been correlated to disease but it is also a normal resident of the mucus layer [49], [50]–[53] and skeleton [53], [54]. The bacterium has also been isolated from the water column surrounding coral colonies indicating that there may be a specific interaction of the bacterium with the coral [49], [52]. The effect of the bacterium infectivity on coral nubbins was density dependent with further challenge experiments showing that an inoculation of 10^6^ CFU/ml caused disease signs whereas dilutions of 10^5^ and 10^4^ did not. This effect has been documented in the larvae of the oyster, *Crassostrea gigas* where a decreased *Alteromonas* sp. inocula causes a delayed effect in infectivity progression [84].

Correspondence analysis of bacterial profiles generated from DGGE analysis demonstrated that the *Alteromonas* sp. challenged corals caused a major shift in the coral associated bacterial community away from a stable community observed in control nubbins. This shift was likely the result of necrosing tissue allowing colonization of many other opportunistic bacteria, potentially enhancing a stronger immune response by the coral. Previous studies have shown similar shifts in the microbial communities during disease and bleaching events [85]. The *V. coralliilyticus* challenged corals also demonstrated a shift in the bacterial community from its experimental control, though the resultant community profile was similar to the control group of the *Alteromonas* sp. treatment. This result further supports the notion that the bacterial community shift was unlikely to have a detrimental effect on coral fitness.

Our findings verify, at the transcriptional level, the functional involvement of C3–like protein and Hsp70 in the immune response of *A. millepora* to bacterial challenges. Interestingly, this is the first study that reports the involvement of a heat shock protein in the coral immune response. Further studies investigating whether these genes have a similar role in other coral species are required along with the characterization and confirmation of the functional role of these and other putative immune homolog genes identified in the context of coral immune response. The derived information is of fundamental importance as functional immune genes may be used as bioindicators to assess coral health status.

## Supporting Information

Figure S1 Photographs of *Acropora millepora* coral nubbins, from the second set of experiments, at 48 hours after inoculation with *Vibrio coralliilyticus* at different concentrations. A: 10^4^ CFU/ml; B: 10^5^ CFU/ml; C: 10^6^ CFU/ml; and D: control.
(EPS)

Figure S2 Gel-banding profiles of 16S rDNA-PCR-DGGE of the bacterial communities associated with *Acropora millepora* coral nubbins at 24 h after bacterial inoculation. *Alteromonas* sp. (A); *Vibrio coralliilyticus* (V), and controls (C). Green arrows indicate bands most closely related to *Endozoicomonas sp.* and orange arrows to *Oceanospirillum beijerinckii*.
(EPS)
